# The prognostic value of the central location of pulmonary nodules in osteosarcoma patients

**DOI:** 10.1007/s12672-025-02446-x

**Published:** 2025-05-09

**Authors:** Gehad Ahmed, Maged Elshafiey, Marwa Romeih, Ahmed Kamel, Ahmed Elgammal, Asmaa Salama, Yomna AboTabl, Mohammad Taher

**Affiliations:** 1 Department of General Surgery, Faculty of Medicine, Helwan University, Cairo, Egypt; 2 Department of Surgical Oncology, National Cancer Institute, Cairo University, Cairo, 11976 Egypt; 3 Department of Radio Diagnosis, Faculty of Medicine, Helwan University, Cairo, Egypt; 4 Department of Pediatric Oncology, National Cancer Institute, Cairo University, Cairo, Egypt; 5 Department of Pathology, National Cancer Institute, Cairo University, Cairo, Egypt; 6 Department of Clinical Research, Children’s Cancer Hospital Egypt (CCHE, 57357), Cairo, Egypt; 7 Children’s Cancer Hospital Egypt (CCHE, 57357), Cairo, Egypt

**Keywords:** Osteosarcoma, Central metastasis, Pulmonary metastasectomy, Prognostic factors

## Abstract

**Background:**

Most metastatic osteosarcoma pulmonary nodules exist in the periphery of the lung, while other lesions are close to the airway and vascular branches in the center. Central lesions require anatomical resection. However, there is a scarcity of data on the prognostic value of the central location of pulmonary metastasis in osteosarcoma.

**Methods:**

We reviewed the medical records of osteosarcoma patients with metastasectomy between January 2009 and June 2022. The central lesions were identified as lesions requiring lobectomy or segmentectomy. We divided our cohort into Group A (peripheral lesion) and Group B (central lesion). The primary outcome measures were overall survival (OS) and postmetastasectomy event-free survival (PMEFS).

**Results:**

We identified a total of 204 osteosarcoma patients with pulmonary metastases. Among them, 162 patients underwent metastasectomy: Group A (n = 128, 80.6%) and Group B (n = 34, 19.4%). Group A had a 5-year overall survival rate (OS) of 36.6% ± 5.7%, whereas Group B had a 5-year overall survival rate of 17.5% ± 8.5%, with (p = 0.048). The 3-year PMEFS for peripheral and central lesions was 25.7 ± 5.1 and 11.8 ± 7.1, respectively, with (p = 0.034).

**Conclusion:**

In Osteosarcoma patients who underwent metastasectomy, central pulmonary nodules are associated with lower OS and PMEFS. We recommend, however, to keep offering these patients the option of metastasectomy as a predictor of survival until a more effective treatment is approved.

## Introduction

Osteosarcoma is the most prevalent bone cancer in children and adolescents. The metastatic spread is heterogeneous, with synchronous or metachronous lung metastasis developing in 50% of patients with osteosarcoma[1, 2]. Macroscopic metastasis is the most significant predictor of survival in osteosarcoma patients[3, 4]. The mean 5-year overall survival rate for patients with pulmonary metastases is less than 30% [5, 6]. Surgical resection proved to prolong survival in patients with pulmonary metastases. Lung parenchyma-sparing and achieving a negative margin is crucial when planning for pulmonary resections. This plan depends mainly on the number of metastases, location, and size of the pulmonary nodules.

The majority of metastatic osteosarcoma pulmonary nodules exist in the periphery of the lung, while other lesions are close to the airway and vascular branches in the center. Central lesions necessitate anatomical resection (lobectomy or segmentectomy) [7]. However, prognostic factors associated with improved survival after resecting pulmonary metastasis remain undetermined. In addition, there is a dearth of research regarding the prognostic value of peripheral vs. central location of pulmonary metastasis in osteosarcoma. Therefore, in this study, we examined a homogenous group of metastatic osteosarcoma patients who underwent metastasectomy in order to enhance our understanding of the prognostic effect of the location of metastatic lung nodules.

## Patients and methods

### Methodology

The medical records of all patients with osteosarcoma who underwent metastasectomy between January 2009 and June 2022 at our tertiary center [Children’s Cancer Hospital Egypt (CCHE, 57357)] were reviewed after receiving approval from our Institutional Review Board (number:68/2022, date: 15/12/2022). We collected data including patients’ demographics, tumor characteristics, operative details, follow-up, and length of survival. Characteristics evaluated included tumor laterality, number of metastasectomies, number of resected positive pulmonary metastases (< 5 vs. ≥ 5), the timing of pulmonary metastases (initial vs. delayed), location of pulmonary metastases, resection margin, and response to chemotherapy (good when tumor necrosis ≥ 90% and poor if tumor necrosis < 90% on the metastatic nodules).

Central lesions were determined using a CT scan. We defined them as those lesions abutting a first and/ or second-degree blood vessel or bronchus. In addition, resecting such central lesion would require resectioning that structure (lobectomy or segmentectomy). We divided our cohort into Group A (peripheral lesion) and Group 2 (central lesion). Metastasectomies were performed as radical procedures with curative intents, and no surgery was done on a palliative basis. The eligibility criteria for metastatic lesion operation were no disease other than those affecting the lung and achieving complete resection with negative margins. Several patients in this study underwent multiple thoracotomies. The criteria for reoperation on metastatic lesions were identical to the previous criteria.

Inclusion criteria were patients with metastatic osteosarcoma who underwent metastasectomy and had available CT scans within two weeks of surgery. A radiologist blinded to the outcome reviewed the CT scans of each procedure, and the lesions were classified as central or peripheral. The primary outcome measures were overall survival (OS) and postmetastasectomy event-free survival (PMEFS).

### Statistical analysis

Group characteristics were compared using t-tests (means) or chi-square tests (proportions/categorical variables). Survival curves were made using the Kaplan–Meier actuarial survival method, and statistical significance was determined utilizing log-rank testing. Multiple survival analyses using the Cox proportional hazards model of all prognostic factors other than the factor of study concern (location of lung nodule) were done using the backwards conditional method, and after this we added the factor of study concern (the location of the lung nodule) to the model in SPSS version 22.0. Two-sided p-values lower than 0.05 were settled to be significant. For this analysis, the PMEFS was determined from the date of metastasectomy to the date of an event, which could be relapse, death, or last follow-up. The overall survival was specified as the duration from the pathological diagnosis until death or last follow-up.

## Results

During the study period, our hospital received 581 patients with osteosarcoma; of them, 204 patients developed pulmonary metastases either at presentation or later. Among those with pulmonary metastasis, 162 underwent pulmonary metastasectomy, with a total of 263 thoracotomies. These 162 patients represent our cohort for analysis. The median age of the subjects at the time of the procedure was 13 years, ranging from 3.6 to 18.3 years. There were (n = 83, 51%) males and (n = 79, 49%) females. The median follow-up duration was 48.9 months, ranging from 12 to 153.3 months. Additionally, 59 (37%) of patients underwent only one thoracotomy, and (n = 103, 63%) underwent two or more thoracotomies for relapse. The majority of the patients (n = 128, 80%) were in Group A, while Group B included the remaining subjects (n = 34, 20%), as shown in Table 1. Of the 42 patients who did not undergo surgery, 14 had central nodules, and 28 had peripheral nodules. The inoperability was due to the presence of other systemic metastasis and/or progressive disease while on chemotherapy.

**Table 1:** Patients and tumor characteristics

Patients characteristics	Number (n = 162)	Percentage %
Laterality		
Unilateral	102	63%
Bilateral	60	37%
Number of nodules		
< 5	112	69.1%
≥ 5	50	30.9%
Tumor Necrosis (response to chemotherapy)		
Good response (necrosis ≥ 90%)	44	27.2%
Poor response (necrosis < 90%)	118	72.8%
Timing of metastasis		
Initial	68	42%
Delayed	94	58%
Location		
Peripheral	128	79%
Central	34	21%
Procedure (n = 263)		
Wedge resection	210	79.8%
Segmentectomy	28	10.6%
Lobectomy	25	9.6%
Total number of procedures		
Central	40	15.2%
Peripheral	223	84.8%

There was no statistically significant difference in patients and tumor characteristics between the two groups, as depicted in Table 2. Central nodules were resected in 34 patients through 40 procedures, and peripheral nodules were resected in 128 Patients through 223 procedures.

**Table 2:** Patients & tumor characteristics in relation to the location of pulmonary nodules

Patients characteristics	Central (n = 34)	Peripheral (n = 128)	P value
Age (years)	14 ± 2.9 (4.2–16)	12.4 ± 3.2 (3.6–21)	0.082
Laterality			
Unilateral	21 (61.8%)	81 (63.3%)	0.87
Bilateral	13 (38.2%)	47 (36.7%)	
Number of nodules			
< 5	23 (67.6%)	89 (69.5%)	0.833
≥ 5	11 (32.4%)	39 (30.5%)	
Tumor necrosis (response to chemotherapy)			
Good response (necrosis ≥ 90%)	9(20.9%)	40 (31.2%)	0.590
Poor response (necrosis < 90%)	25 (79.1%)	88 (68.8%)	
Timing of metastasis			
Initial	14 (58.8%)	54 (42.2%)	0.91
Delayed	20 (41.2%)	74 (57.8%)	

**Fig. 1:**
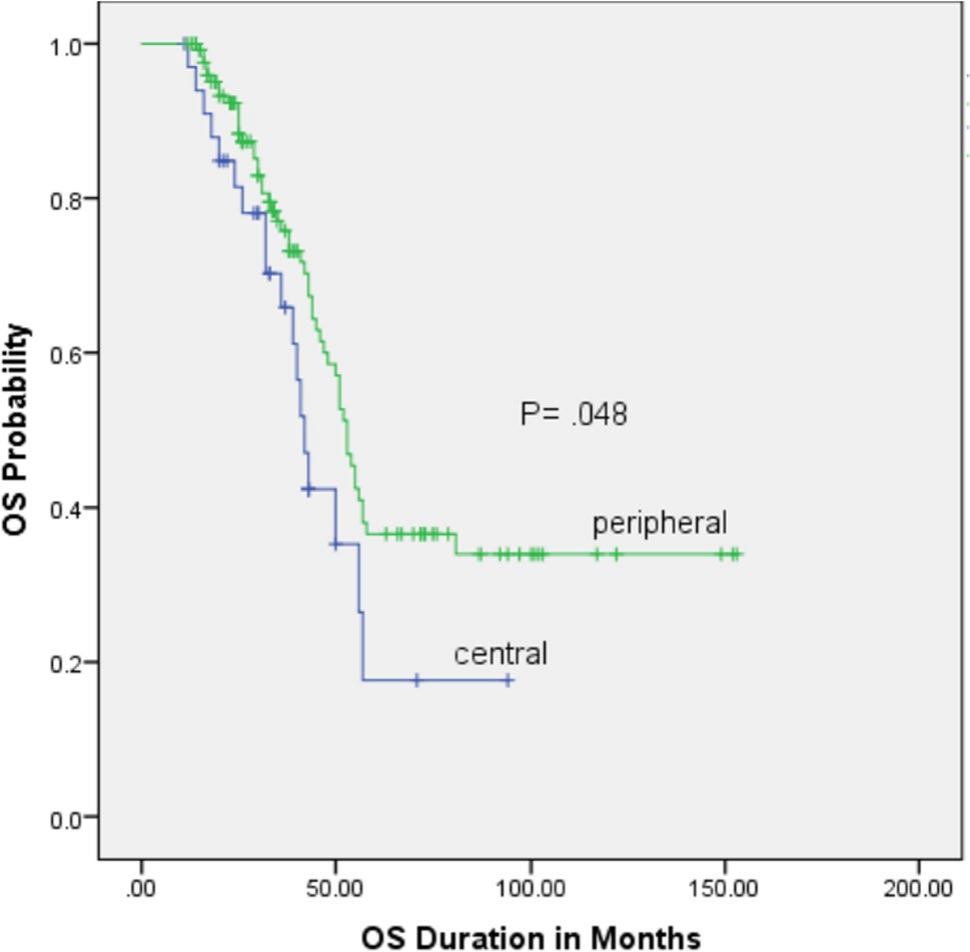
Overall Survival (OS) in central vs peripheral metastatic nodules

**Fig. 2:**
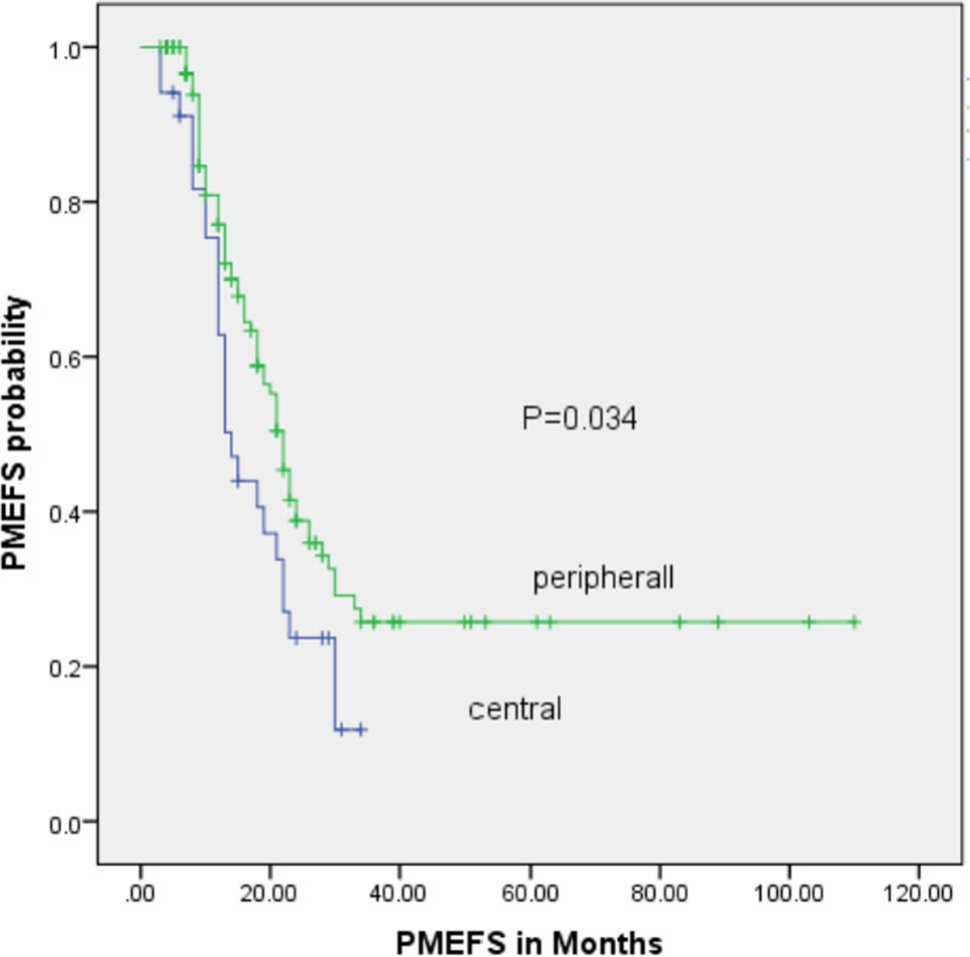
Postmetstasectomy event-free survival (PMEFS) in central vs peripheral metastatic nodules

The whole group's 5-year OS and 3-year PMEFS were 32.7 ± 4.7 and 22.5 ± 4.3, respectively. The estimated 5-year OS probability of groups A and B were 36.6 ± 5.7% and 17.6 ± 8.5% respectively (p = 0.043) (Fig. 1). The 3-year PMEFS probability of groups A and B were 25.7 ± 5.1 and 11.8 ± 7.1, respectively (p = 0.034) (Fig. 2). The simple analysis revealed that the factors associated with poor OS were the central location of lung nodules, bilateral metastasis, and poor response to chemotherapy. Other factors, such as the number of nodules and timing of metastasis, were not associated with survival, as shown in Table 3. The multiple analysis using backward conditional analysis, including all prognostic factors except the location of lung nodule, indicated that laterality (p-value 0.042) and response to chemotherapy (p-value 0.009) were statistically significant, and when added the location of lung nodule to the model the central location and response to chemotherapy were the only significant factors (Table 4).

**Table 3:** Simple analysis of the risk factors associated with 5-year overall survival (OS) & postmetastasectomy event-free survival (PMEFS)

Prognostic factor	5-year OS ± SE	p-value	3-year PMEFS ± SE	p-value
Laterality				
Unilateral	40.6 ± 6.2	0.044*	26.1 ± 5.6	0.102
Bilateral	14.2 ± 6.2		15.5 ± 6.8	
Number of nodules				
< 5	32.8 ± 6.1	0.36	25.2 ± 5.3	0.172
≥ 5	30.7 ± 9.4		15.7 ± 7.3	
Tumor necrosis (response to chemotherapy)				
Good response (necrosis ≥ 90%)	37.1 ± 6	0.011*	22.6 ± 8.2	0.183
Poor response (necrosis < 90%)	20.8 ± 2.6		16.5 ± 5.7	
Timing				
Initial	25.4 ± 7.5	0.051	18.9 ± 6.1	0.298
Delayed	37.5 ± 6.5		25.2 ± 5.3	
Location				
Peripheral	36.6 ± 5.7	0.043*	25.7 ± 5.1	0.034*
Central	17.6 ± 8.5		11.8 ± 7.1	

SE: standard error, *: indicates a significant p-value

**Table 4:** Multiple analysis of the risk factors associated with 5-year overall survival (OS) & postmetastasectomy event-free survival (PMEFS)

Prognostic factor	5-year OS		3-year PMEFS	
	Hazard ratio (95% CI)	p -value	Hazard ratio (95% CI)	p- value
Laterality				
Unilateral	1 (Ref)	0.391	1 (Ref)	0.133
Bilateral	1.28 (0.72–2.25)		1.33 (0.89–2.30)	
Number of nodules				
< 5	1 (Ref)	0.25	1 (Ref)	0.683
≥ 5	1.37 (0.79–2.38)		1.11 (0.66–1.84)	
Tumor necrosis (response to chemotherapy)				
Good response (necrosis ≥ 90%)	1 (Ref)	0.024*	1 (Ref)	0.702
Poor response (necrosis < 90%)	1.76 (1.078–2.87)		1.02 (0.923–1.12)	
Timing of metastasis				
Delayed	1 (Ref)	0.22	1 (Ref)	0.259
Initial	1.407(0.812–2.43)		1.28 (0.83–1.95)	
Location				
Peripheral	1 (Ref)	0.027*	1 (Ref)	0.029*
Central	1.87 (1.072–1.87)		1.69 (1.05–2.71)	

CI: confidence interval, Ref: reference, *: indicates a significant p-value

The 3-year PMEFS of the whole group was 22.2 ± 4.3. In Group A, there were 66 relapses, with 8 of them being systemic relapses (12%). There were 26 relapses in Group B, with 14 cases (54%) being systemic relapses. The simple analysis revealed that the only factor associated with poor PMEFS is the central location of pulmonary (p = 0.034). It also retained its significance in the multiple analysis (HR = 1.69). The simple and multiple analyses revealed that factors such as response to chemotherapy, number of nodules, and laterality were not associated with PMEFS (Tables 3, 4).

## Discussion

The lung is the primary site of osteosarcoma metastases, with only 10–15% of pulmonary metastases located in central regions and the majority in peripheral areas [7]. Studies have demonstrated that surgical resection and the absence of extrapulmonary metastasis enhance survival rates in patients with pulmonary metastases [8]. Other prognostic factors associated with improved survival after resecting pulmonary metastasis remain undetermined [9, 10].

In this study, we examined the prognostic significance of the location of pulmonary nodules in osteosarcoma patients. To eliminate the confounding effect of the more established prognostic factors, namely, resection of metastasis and presence of extrapulmonary metastasis, we selected a relatively homogenous group of patients with osteosarcoma with the lung as the only site of metastasis who had metastasectomy. Our results suggest that the central location of pulmonary nodules is one of the factors significantly associated with poor OS and PMEFS in both simple and multiple analysis. Other factors (number of lung nodules, laterality, and timing of lung metastasis) were not associated with survival.

In the present study, the 5-year OS of the whole cohort was 32.7 ± 4.7%, which is consistent with previous studies that reported 5-year OS in the range of 30% [11–13]. The independent factors shown to predict long-term survival after pulmonary metastasectomy in the present study were the central location of the pulmonary nodule, bilaterality of lung metastasis, and response to chemotherapy. However, in multiple analysis, central location (HR = 1.87) and response to chemotherapy (HR = 1.76) were the only significant factors.

Only a limited number of studies have examined the prognosis of osteosarcoma with central metastatic lung nodules. Matsubara and colleagues reported that 11% of metastasectomies were performed for central metastasis[14]. This finding agrees with our study in which surgery for central lesions represents (40/264, 15%) of all procedures.

In the present study, the probability of 5-year OS of central lesions was 17.6%. This result is higher than those reported by Letourneau et al., who demonstrated a poor prognosis for central nodules after surgery. In addition, there was only a 7% probability of 3-year survival [7]. They determined that surgery was not a viable choice for these patients and recommended that another line of treatment should be discussed with the patients. We partially disagree with this conclusion despite that, in our study, patients with central metastasis who underwent metastasectomy via lobectomy or segmentectomy had significantly lower survival rates than those with peripheral lesions. We believe that metastasectomy is the only treatment option; thus, we recommend metastasectomy for those patients as the most viable treatment. The poor outcomes reported by Letourneau et al. could be attributed to the small number of patients with heterogeneous risk factors and the exclusion of numerous crucial prognostic factors from the analysis. We think that the poor survival rates for central lesions compared to peripheral locations can be partially explained by the fact that most of the relapses after resection of central lesions were systemic outside the lung. In contrast, in peripheral lesions, relapses were predominantly localized to the lung and could be addressed by surgery.

Prior research on osteosarcoma has shown inconsistent findings regarding prognostic factors linked to survival following the removal of metastatic osteosarcoma. Most of these reports also ignored the location of pulmonary nodules in the analysis. Bacci et al. and other multicentral studies demonstrated that incomplete resection, greater numbers of pulmonary nodules, and bilaterality of metastatic nodules were independent prognostic factors for poor survival after metastasectomy [15, 16]. Other researchers did not validate these prognostic factors. They determined that resection margins, number of pulmonary nodules, disease bilaterality, and disease-free interval do not significantly affect survival [11, 14, 17]. The discrepancies in findings among various reports can be attributed to the retrospective study design of all the reports, the rarity of the disease, varying inclusion criteria, and a relatively small number of patients in different studies, which could impede multiple analysis.

The 3-year PMEFS for the group that underwent lung relapse surgery was 22.5% in the current study. This number is greater than the one reported by Leary et al., who stated an 11.8% chance of post-relapse-free survival for all relapse sites. Our study found that the central location was the sole factor of PMEFS in osteosarcoma patients in both simple and multiple analyses. Our results partially align with those of Kim et al., who reported that metastasectomy was the most significant prognostic factor, and poor histologic response to primary chemotherapy and site of metastasis were independent negative prognostic factors. Their report also predicts postmetastasectomy survival (PMS) by considering the total number of negative factors [17]. The 5-year PMS for patients without any negative prognostic factors is estimated at 60.2%. For those with one factor, it is 31.6%, and for those with more than two factors, it is 3.6% [17]. Leary et al. identified the factors linked to post-relapse event-free survival (PREFS) as the time of relapse, the likelihood of achieving complete remission, and treatment received at first relapse [18].

The current study has some limitations, such as the small number of patients with central metastatic nodules. Therefore, we could not conduct a statistical analysis of factors associated with survival among this group. Besides this, its retrospective nature is another limitation.

## Conclusion

In osteosarcoma patients who underwent metastasectomy, central pulmonary nodules are linked to reduced overall survival and postmetastasectomy event-free survival. We recommend, however, to keep offering these patients the option of metastasectomy as a predictor of survival until a more effective treatment is approved.

## Data Availability

Data are presented in the study, and any additional data are available from the corresponding author upon request.

## Abbreviations

OS: Overall survival
PMEFS: Post-metastasectomy event-free survival
PMS: Post-metastasectomy survival
PREFS: Post-relapse event-free survival

## References

[1] Wu PKPK, Chen WM, Chen CF, Lee OK, Haung CK, Chen TH, et al. Survival of pediatric patients after relapsed osteosarcoma: the St. Jude Children’s Research Hospital experience. J Clin Oncol. 2015;104:194–9. 10.1002/cncr.28111. 10.1002/cncr.28111 PMC4273500 23625626

[2] Vasquez L, Tarrillo F, Oscanoa M, Maza I, Geronimo J, Paredes G, et al. Analysis of prognostic factors in high-grade osteosarcoma of the extremities in children: a 15-year single-institution experience. Front Oncol. 2016;6:22. 10.3389/fonc.2016.00022. 26904501 10.3389/fonc.2016.00022 PMC4745606

[3] Smeland S, Bielack SS, Whelan J, Bernstein M, Hogendoorn P, Krailo MD, et al. Survival and prognosis with osteosarcoma: outcomes in more than 2000 patients in the EURAMOS-1 (European and American Osteosarcoma Study) cohort. Eur J Cancer. 2019;109:36–50. 10.1016/J.EJCA.2018.11.027. 30685685 10.1016/j.ejca.2018.11.027 PMC6506906

[4] Bielack SS, Kempf-Bielack B, Delling G, Exner GU, Flege S, Helmke K, Bielack SS, Kempf-Bielack B, Delling G, et al. Prognostic factors in high-grade osteosarcoma of the extremities or trunk: an analysis of 1,702 patients treated on Neoadjuvant Cooperative Osteosarcoma Study Group Protocols. J Clin Oncol. 2017;20:776–90. 10.1200/JCO.2002.20.3.776. 10.1200/JCO.2002.20.3.776 11821461

[5] Kager L, Zoubek A, Pötschger U, Kastner U, Flege S, Kempf-Bielack B, et al. Primary metastatic osteosarcoma: presentation and outcome of patients treated on neoadjuvant Cooperative Osteosarcoma Study Group protocols. J Clin Oncol. 2003;21:2011–8. 10.1200/JCO.2003.08.132. 12743156 10.1200/JCO.2003.08.132

[6] Daum R, Roth H, Zachariou Z. Tumor Infiltration of the Vena Cava in Nephroblastoma. Eur J Pediatr Surg. 1994;4:16–20. 10.1055/S-2008-1066059/BIB. 8199126 10.1055/s-2008-1066059

[7] Letourneau PA, Xiao L, Harting MT, Lally KP, Cox CS, Andrassy RJ, et al. Location of pulmonary metastasis in pediatric osteosarcoma is predictive of outcome. J Pediatr Surg. 2011;46:1333–7. 10.1016/J.JPEDSURG.2010.12.013. 21763830 10.1016/j.jpedsurg.2010.12.013 PMC3768269

[8] Pastorino U, Buyse M, Friedel G, Ginsberg RJ, Girard P, Goldstraw P, et al. Long-term results of lung metastasectomy: prognostic analyses based on 5206 cases. J Thorac Cardiovasc Surg. 1997;113:37–49. 10.1016/S0022-5223(97)70397-0. 9011700 10.1016/s0022-5223(97)70397-0

[9] Ahmed G, Elshafiey M, Romeih M, Elgammal A, Kamel A, Salama A, et al. Prognostic significance of the ratio of surgically resected to radiologically detected lung nodules in patients with metastatic osteosarcoma. Surg Oncol. 2022;40: 101701. 10.1016/J.SURONC.2021.101701. 34992029 10.1016/j.suronc.2021.101701

[10] Juergens H, Berdel W, Kempf-Bielack B, Bielack SS, Jürgens H, Branscheid D, et al. Osteosarcoma relapse after combined modality therapy: an analysis of unselected patients in the cooperative osteosarcoma study group (COSS). Artic J Clin Oncol. 2005;23:559–68. 10.1200/JCO.2005.04.063. 10.1200/JCO.2005.04.063 15659502

[11] Harting MT, Blakely ML, Jaffe N, Cox CS, Hayes-Jordan A, Benjamin RS, et al. Long-term survival after aggressive resection of pulmonary metastases among children and adolescents with osteosarcoma. J Pediatr Surg. 2006;41:194–9. 10.1016/J.JPEDSURG.2005.10.089. 16410132 10.1016/j.jpedsurg.2005.10.089

[12] Huang Y-M, Hou C-H, Hou S-M, Yang R-S. The metastasectomy and timing of pulmonary metastases on the outcome of osteosarcoma patients. Clin Med Oncol. 2009. 10.4137/CMO.S531/ASSET/IMAGES/LARGE/10.4137_CMO.S531-FIG2.JPEG. 20689616 10.4137/cmo.s531 PMC2872604

[13] Wu PK, Chen WM, Chen CF, Lee OK, Haung CK, Chen TH. Primary osteogenic sarcoma with pulmonary metastasis: clinical results and prognostic factors in 91 patients. Jpn J Clin Oncol. 2009;39:514–22. 10.1093/JJCO/HYP057. 19525290 10.1093/jjco/hyp057

[14] Matsubara E, Mori T, Koga T, Shibata H, Ikeda K, Shiraishi K, et al. Metastasectomy of pulmonary metastases from osteosarcoma: prognostic factors and indication for repeat metastasectomy. J Respir Med. 2015;2015:1–5. 10.1155/2015/570314.

[15] Bacci G, Rocca M, Salone M, Balladelli A, Ferrari S, Palmerini E, et al. High grade osteosarcoma of the extremities with lung metastases at presentation: treatment with neoadjuvant chemotherapy and simultaneous resection of primary and metastatic lesions. J Surg Oncol. 2008;98:415–20. 10.1002/JSO.21140. 18792969 10.1002/jso.21140

[16] Briccoli A, Rocca M, Salone M, Bacci G, Ferrari S, Balladelli A, et al. Resection of recurrent pulmonary metastases in patients with osteosarcoma. Cancer. 2005;104:1721–5. 10.1002/CNCR.21369. 16155943 10.1002/cncr.21369

[17] Kim W, Han I, Lee JS, Cho HS, Park JW, Kim HS. Postmetastasis survival in high-grade extremity osteosarcoma: a retrospective analysis of prognostic factors in 126 patients. J Surg Oncol. 2018;117:1223–31. 10.1002/JSO.24963. 29409122 10.1002/jso.24963

[18] Leary SES, Wozniak AW, Billups CA, Wu J, McPherson V, Neel MD, et al. Survival of pediatric patients after relapsed osteosarcoma: the St. Jude Children’s Research Hospital experience. Cancer. 2013;119:2645–53. 10.1002/CNCR.28111. 23625626 10.1002/cncr.28111 PMC4273500

